# Characterization of Antinuclear, Myositis-Specific, and Myositis-Associated Antibodies in a Large Sample of Patients With Idiopathic Inflammatory Myopathies

**DOI:** 10.7759/cureus.104553

**Published:** 2026-03-02

**Authors:** Rafaella A Barbosa, Samuel K Shinjo

**Affiliations:** 1 Division of Rheumaotlogy, Faculdade de Medicina da Universidade de São Paulo (FMUSP), São Paulo, BRA; 2 Division of Rheumatology, Faculdade de Medicina da Universidade de São Paulo (FMUSP), São Paulo, BRA

**Keywords:** antinuclear antibody, autoantibody, immune myositis, systemic autoimmune disease, systemic autoimmune myopathies

## Abstract

Background: Antinuclear antibody (ANA) testing is routinely employed for screening systemic autoimmune diseases due to its accessibility and practicality. However, its diagnostic and clinical significance in idiopathic inflammatory myopathies (IIMs) remains uncertain. Therefore, this study aimed to determine the frequency and distribution of ANA, myositis-specific autoantibodies (MSAs), and myositis-associated autoantibodies (MAAs) in IIMs and to explore their interrelationships.

Methods: In this single-center study, adult patients with definite IIMs were included. ANA was assessed by indirect immunofluorescence on HEp-2 cells, and MSA/MAA was analyzed using a commercial line blot assay, both performed at baseline.

Results: Among 580 screened patients, 229 were excluded due to cancer-associated myositis (N=45), overlap myositis (N=21), unavailable serum samples (N=142), or missing ANA data (N=21). The final cohort included 351 patients, of whom 261 (74.4%) were ANA-positive (titers 1:80-1:1280). The predominant ANA pattern was isolated nuclear (61.7%), followed by cytoplasmic (18.0%) and nucleolar (6.9%). Multiple ANA patterns were observed in 13.4% of cases, while distinct patterns were identified in 34.1%. MSA and MAA were more frequently detected in ANA-positive patients, although they were also present in ANA-negative individuals.

Conclusions: ANA positivity was observed in approximately three-quarters of IIM patients, exhibiting heterogeneous immunofluorescence patterns across subtypes. Although MSA and MAA were more common among ANA-positive individuals, their occurrence in ANA-negative patients highlights the necessity of combined testing approaches. These findings underscore the complementary roles of ANA and MSA/MAA assays in the comprehensive immunological evaluation of IIMs.

## Introduction

Idiopathic inflammatory myopathies (IIMs), also referred to as systemic autoimmune myopathies, are rare rheumatic diseases that primarily affect skeletal muscle but may also involve the skin, lungs, heart, and joints [1]. These conditions are classified into distinct subtypes based on clinical, laboratory, histopathological, and prognostic features, including dermatomyositis (DM), clinically amyopathic dermatomyositis (CADM), polymyositis (PM), immune-mediated necrotizing myopathy (IMNM), and anti-synthetase syndrome (ASyS) [1,2].

From an immunological perspective, IIMs are characterized by the presence of myositis-specific autoantibodies (MSAs) and myositis-associated autoantibodies (MAAs) [1-3]. MSAs are highly specific to IIM and include anti-Mi-2, -MDA-5 (Melanoma Differentiation-Associated Gene 5), -TIF-1γ (Transcription Intermediary Factor 1-Gamma), -SAE (Small Ubiquitin-Like Modifier Activating Enzyme), -NXP-2 (Nuclear Matrix Protein 2), -SRP (Signal Recognition Particle), and -HMGCR (3-Hidroxy-3-Methylglutaryl-Conezyme A Reductase) antibodies, as well as anti-aminoacyl-tRNA synthetases (anti-ARS: anti-Jo-1, -OJ, -EJ, -PL-7, -PL-12, -KS, -Zo, -Ha, -Cys, and -Val) [1-4]. In contrast, MAAs can be found not only in patients with IIM but also in other connective tissue diseases, such as those with anti-Ro-52, -PM/Scl, and -Ku antibodies [1-4].

The antinuclear antibody (ANA) test, also referred to as the antinuclear factor test, represents a key biomarker of systemic autoimmunity. ANA testing enables the detection of a broad spectrum of autoantibodies directed against diverse intracellular antigens, providing information on antibody titers and characteristic immunofluorescence patterns, such as homogeneous nuclear, fine-speckled cytoplasmic, and homogeneous nucleolar, among others [5,6].

The interpretation of a positive ANA result, in conjunction with clinical manifestations, can aid in identifying potential autoantibody profiles and in supporting the diagnosis of autoimmune diseases [5,7]. In patients with IIMs, ANA pattern analysis may provide additional insights into the underlying autoimmune process. Furthermore, ANA testing is widely accessible and cost-effective compared with specific autoantibody assays.

Despite its extensive clinical use, the relationship between ANA patterns, MSAs, MAAs, clinical manifestations, and IIM subtypes remains insufficiently explored. Many available studies have methodological limitations, such as reliance on the historical Bohan and Peter criteria for case definition [8], inclusion limited to patients with interstitial lung disease (ILD) [9], or restriction to particular IIM subtypes, such as DM [10], ASyS [11], or IMNM [12].

Therefore, this study aimed to (i) determine the frequency of ANA, MSA, and MAA positivity; (ii) analyze associations between ANA immunofluorescence patterns and IIM subtypes; and (iii) evaluate the co‑occurrence of ANA with specific MSA and MAA profiles in adult patients with IIMs.

## Materials and methods

This was a single-center retrospective cohort study conducted between July 2001 and July 2025, including adult patients with IIMs who were treated at our tertiary care center. The study was approved by our institutional ethics committee (CAAE 34954620.5.0000.0068).

Patients with PM, DM, and CADM were classified according to the 2017 European League Against Rheumatism/American College of Rheumatology (EULAR/ACR) criteria for IIMs [13]. ASyS was defined according to Behrens Pinto et al. [14], and IMNM according to the European Neuromuscular Centre (ENMC) International Workshop criteria [15].

Patients with non-autoimmune myopathies, those with myopathies associated with other systemic autoimmune diseases (e.g., rheumatoid arthritis, systemic sclerosis, systemic lupus erythematosus, or Sjögren’s disease), neoplasm-associated myopathies, incomplete clinical data, or unavailable serum samples were excluded.

Clinical data were obtained from electronic medical records containing pre-standardized and structured information, including (i) Demographic variables: age at diagnosis and sex; (ii) Clinical manifestations: disease onset, follow-up duration, cutaneous manifestations (Gottron’s papules or sign, heliotrope rash, “mechanic’s hands”), Raynaud’s phenomenon, systemic manifestations (fever, arthritis), and proximal and distal muscle weakness graded according to the Medical Research Council (MRC) scale [16]; (iii) Laboratory data: ANA results and serum levels of muscle enzymes (creatine phosphokinase, aspartate aminotransferase, alanine aminotransferase, and lactate dehydrogenase); (iv) Complementary investigations: pulmonary abnormalities identified by high-resolution computed tomography, including “ground-glass” opacities and/or pulmonary fibrosis.

Serum samples (15 mL of blood) were collected at baseline and stored at - 20°C in the laboratory biorepository. MSA and MAA were analyzed using a commercial line blot assay (Euroimmun, Lübeck, Germany) for anti-Mi-2, -MDA-5, -TIF-1γ, -SAE, -NXP-2, -SRP, -Jo-1, -OJ, -EJ, -PL-7, -PL-12, -PM/Scl-75, -PM/Scl-100, -Ro-52, and -Ku antibodies. The most recent generation of the assay, Autoimmune Inflammatory Myopathies 16 Ag (IgG), allows quantification of all these autoantibodies. The previous-generation kit, EUROLINE Myositis Antigen Profile 3 (IgG), detects the same set of markers, except for anti-MDA‑5, -NXP‑2, -SAE, and -TIF-1γ. Reactivity was semi-quantitatively classified as negative (0/+++), weakly positive (+/+++), moderately positive (++/+++), or strongly positive (+++/+++) by two independent investigators. Only moderate or strong bands were considered positive, according to a previously validated protocol [2]. Importantly, neither the Autoimmune Inflammatory Myopathies 16 Ag (IgG) nor the EUROLINE Myositis Antigen Profile 3 (IgG) panels include anti‑HMGCR, and this autoantibody was therefore not assessed in this cohort.

ANA testing was performed by indirect immunofluorescence on Hep-2 cells by a specialized clinical immunologist with more than five decades of experience in ANA interpretation. ANA testing was performed at a screening dilution of 1:80, with positivity defined as titers ≥1:80. ANA patterns were assigned according to the International Consensus on ANA Patterns (ICAP) criteria. Discordant results in the line blot assay were re‑evaluated jointly by both investigators, and consensus or repeat testing was required for adjudication. All sera were stored at -20 °C and analyzed within a maximum of two freeze-thaw cycles.

Statistical analysis

The normality of data distribution was assessed using the Kolmogorov-Smirnov test. Continuous variables were expressed as mean ± standard deviation or median [interquartile range, IQR: 25th-75th], and categorical variables as absolute numbers and frequencies (%). Group comparisons were performed using Student’s t-test or Mann-Whitney U test for quantitative variables, and chi-square or Fisher’s exact test for qualitative variables, as appropriate. A p-value < 0.05 was considered statistically significant. All analyses were performed using GraphPad Prism version 8.0.2 (GraphPad Software, San Diego, California, USA).

## Results

**Figure 1:**
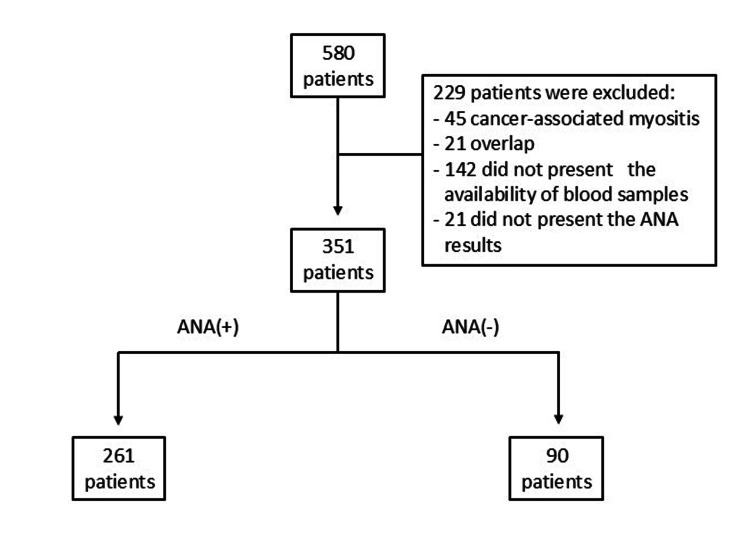
Flowchart of patient selection ANA: antinuclear antibody.

Of the 580 patients with IIMs, 229 (39.5%) were excluded due to unavailable serum samples for autoantibody testing (N=142), association with malignancy (N=45), overlap with other systemic autoimmune diseases (N=21), and/or missing ANA data (N=21). The final cohort therefore comprised 351 patients (Figure 1). Among these, 171 serum samples were analyzed using the EUROLINE Myositis Antigen Profile 3 (IgG), whereas the remaining samples were assessed with the Autoimmune Inflammatory Myopathies 16 Ag (IgG) assay.

Among these, 261 patients (74.4%) were ANA-positive. The frequency of ANA positivity differed significantly across IIM subtypes (P<0.001), being most common, in frequency, in DM (N=121, 82.3%) and PM (N=35, 81.4%), intermediate in ASyS (N=57, 75.0%), and less frequent in IMNM (N=25, 59.5%) and CADM (N=23, 53.5%) (Table 1).

**Table 1:** Distribution of the antinuclear factor according to the subtype of idiopathic inflammatory myopathies, general characteristics of the patients, and the type of myositis-specific and myositis-associated autoantibodies. Data are expressed as median (25th - 75th) or absolute number (%). ANA: antinuclear antibody; ASyS: antisynthetase syndrome; CADM: clinically amyopathic dermatomyositis; DM: dermatomyositis; IIM: idiopathic inflammatory myopathies; IMNM: immune-mediated necrotizing myopathy; PM: polymyositis.

	Total (N=351)	ANA(+) (N=261)	ANA(-) (N=90)	p-value
IIM subtypes				
DM	147 (100)	121 (82.3)	26 (17.7)	<0.001
PM	43 (100)	35 (81.4)	8 (18.6)	
ASyS	76 (100)	57 (75.0)	19 (25.0)	
IMNM	42 (100)	25 (59.5)	17 (40.5)	
CADM	43 (100)	23 (53.5)	20 (46.5)	
Female sex	264 (100)	201 (76.1)	63 (23.9)	0.184
Age at disease diagnosis (years)	44 (32-56)	44 (33-55)	44 (31-58)	0.677
Duration of follow-up (months)	56 (17-116)	57 (20-117)	51 (10-97)	0.151
Autoantibodies				
Myositis-specific	184 (100)	142 (77.2)	42 (22.8)	0.473
Anti-MDA-5	31/171 (100)	21 (67.7)	10 (32.3)	0.422
Anti-Mi-2	29 (100)	25 (86.2)	4 (13.8)	0.184
Anti-Jo-1	51 (100)	34 (66.7)	17 (33.3)	0.245
Anti-EJ	5 (100)	4 (80.0)	1 (20.0)	>0.999
Anti-OJ	2 (100)	1 (50.0)	1 (50.0)	0.450
Anti-PL-7	14 (100)	12 (85.7)	2 (14.3)	0.532
Anti-PL-12	14 (100)	14 (100)	0	-
Anti-SRP	14 (100)	11 (75.6)	3 (21.4)	>0.999
Anti-TIF-1γ	12/171 (100)	10 (83.3)	2 (16.7)	0.738
Anti-SAE	12/171 (100)	10 (83.3)	2 (16.7)	0.738
Anti-NXP-2	9/171 (100)	7 (77.8)	2 (22.2)	>0.999
Myositis-associated	120 (100)	94 (78.3)	26 (21.7)	0.383
Anti-Ro-52	100 (100)	76 (76.0)	24 (24.0)	0.739
Anti-PM/Scl75	14 (100)	12 (85.7)	2 (14.3)	0.532
Anti-PM/Scl100	11 (100)	11 (100)	0	-
Anti-Ku	13 (100)	12 (92.3)	1 (7.7)	0.199
More than one autoantibody	84 (100)	66 (78.6)	18 (21.4)	0.422

No significant differences were found between ANA-positive and ANA-negative groups regarding sex distribution (p=0.184), age at diagnosis (p=0.677), or follow-up duration (p=0.151). Likewise, the frequencies of MSA and MAA did not differ significantly between ANA-positive and ANA-negative groups (p=0.473 and p=0.383, respectively).

Among MSAs, ANA positivity rates varied but did not reach statistical significance in frequencies: anti-Mi-2 (N=25, 86.2%), -MDA-5 (N=21, 67.7%), Jo-1 (N=34, 66.7%), and -SRP (N=11, 75.6%). Among MAAs, ANA positivity was also high, particularly for anti-Ku (N=12,92.3%), although not statistically significant. Anti-PL-12 and -PM/Scl-100 antibodies were detected exclusively in ANA-positive patients.

In the ANA‑positive group (n=261), nuclear, cytoplasmic, and nucleolar patterns were identified, with pattern overlap occurring in a subset of patients; therefore, individual pattern counts exceeded the total number of ANA‑positive individuals (Table 2).

**Table 2:** Distribution of the type of myositis-specific and myositis-associated autoantibodies in patients with dermatomyositis. Data are expressed as an absolute number (%). Ac: antibodies; ANA: antinuclear antibody.

ANA(+) (n=147)	Nuclear (N=110)	Nucleolar (N=12)	Cytoplasmic (N=8)
Myositis-specific Ac			
Anti-MDA-5	11/61 (18.0)	1/6 (16.7)	0
Anti-Mi-2	18 (16.4)	0	1 (12.5)
Anti-Jo-1	2 (1.8)	1 (8.3)	0
Anti-EJ	1 (0.9)	0	0
Anti-OJ	1 (0.9)	0	0
Anti-PL-7	2 (1.8)	1 (8.3)	0
Anti-PL-12	1 (0.9)	0	0
Anti-SRP	2 (1.8)	0	0
Anti-TIF-1γ	9/61 (14.8)	1/6 (16.7)	0
Anti-SAE	8/61 (13.1)	0	0
Anti-NXP-2	5/61 (8.2)	0	0
Myositis-associated Ac			
Anti-Ro-52	22 (20.0)	3 (25.0)	1 (12.5)
Anti-PM/Scl75	3 (2.7)	3 (25.0)	0
Anti-PM/Scl100	3 (2.7)	3 (25.0)	0
Anti-Ku	4 (3.6)	0	0

When IIM subtypes were analyzed according to the ANA pattern, the nuclear pattern was most frequently associated with DM (N=103/184, 56.4%), whereas the cytoplasmic pattern predominated among ASyS patients (N=37/66, 56.1%).

Among ANA-positive DM patients, the nuclear pattern was predominant, and all MSA/MAA were represented, with the highest frequencies observed for antibodies typically associated with DM: anti-MDA-5, -Mi-2, -TIF-1γ, -SAE, and -NXP-2 (Table 2). In ANA-positive CADM patients, the nuclear pattern also predominated, with fewer autoantibodies detected, notably anti-MDA-5, -TIF-1γ, -SAE, and -Ro-52 (Table 3).

**Table 3:** Distribution of the type of myositis-specific and myositis-associated autoantibodies in patients with clinically amyopathic dermatomyositis. Data are expressed as an absolute number (%). Ac: antibodies; ANA: antinuclear antibody.

ANA(+) (n=23)	Nuclear (N=20)	Nucleolar (N=1)	Cytoplasmic (N=3)
Myositis-specific Ac			
Anti-MDA-5	7/17 (41.2)	0	0
Anti-Mi-2	0	0	0
Anti-Jo-1	0	0	0
Anti-EJ	0	0	0
Anti-OJ	0	0	0
Anti-PL-7	0	0	0
Anti-PL-12	0	0	0
Anti-SRP	0	0	0
Anti-TIF-1γ	1/17 (5.9)	0	0
Anti-SAE	2/17(11.8)	0	0
Anti-NXP-2	0	0	0
Myositis-associated Ac			
Anti-Ro-52	3 (15.0)	0	2 (66.7)
Anti-PM/Scl75	0	0	0
Anti-PM/Scl100	0	0	0
Anti-Ku	0	0	0

In PM and IMNM patients, the nuclear pattern was likewise most frequent. Both groups showed reactivity for anti-MDA-5, -Mi-2, -Jo-1, -Ro-52, and -Ku. Additionally, anti-PL-7, -PM/Scl-75, and -PM/Scl-100 antibodies were detected in PM (Table 4), whereas anti-SRP and -NXP-2 were observed in IMNM (Table 5).

**Table 4:** Distribution of the type of myositis-specific, and myositis-associated autoantibodies in patients with polymyositis. Data are expressed as an absolute number (%). Ac: antibodies; ANA: antinuclear antibody.

ANA(+) (n=35)	Nuclear (N=25)	Nucleolar (N=11)	Cytoplasmic (N=9)
Myositis-specific Ac			
Anti-MDA-5	1/7 (14.3)	0	0
Anti-Mi-2	1 (4.0)	2 (18.2)	0
Anti-Jo-1	1 (4.0)	0	0
Anti-EJ	0	0	0
Anti-OJ	0	0	0
Anti-PL-7	1 (4.0)	0	0
Anti-PL-12	0	0	0
Anti-SRP	0	0	0
Anti-TIF-1γ	0	0	0
Anti-SAE	0	0	0
Anti-NXP-2	0	0	0
Myositis-associated Ac			
Anti-Ro-52	6 (24.0)	2 (18.2)	2 (22.2)
Anti-PM/Scl75	5 (20.0)	8 (72.7)	0
Anti-PM/Scl100	4 (16.0)	7 (63.6)	0
Anti-Ku	4 (16.0)	1 (9.1)	0

**Table 5:** Distribution of the type of myositis-specific, and myositis-associated autoantibodies in patients with immune-mediated necrotizing myopathy. Data are expressed as an absolute number (%). Ac: antibodies; ANA: antinuclear antibody.

ANA(+) (n=25)	Nuclear (N=15)	Nucleolar (N=3)	Cytoplasmic (N=9)
Myositis-specific Ac			
Anti-MDA-5	1/8 (12.5)	0	0
Anti-Mi-2	3 (20.0)	0	0
Anti-Jo-1	0	1 (33.3)	0
Anti-EJ	0	0	0
Anti-OJ	0	0	0
Anti-PL-7	0	0	0
Anti-PL-12	0	0	0
Anti-SRP	2 (13.3)	0	6 (66.7)
Anti-TIF-1γ	0	0	0
Anti-SAE	0	0	0
Anti-NXP-2	1/8 (12.5)	0	0
Myositis-associated Ac			
Anti-Ro-52	3 (20.0)	0	3 (33.3)
Anti-PM/Scl75	0	0	0
Anti-PM/Scl100	0	0	0
Anti-Ku	1 (6.7)	0	0

In ASyS, the cytoplasmic ANA pattern predominated. The most frequently detected autoantibodies were those characteristic of the syndrome: anti-Jo-1, -EJ, -PL-7, and -PL-12, as well as anti-Mi-2, -SRP, -NXP-2, -Ro-52, and -Ku (Table 6).

**Table 6:** Distribution of the type of myositis-specific, and myositis-associated autoantibodies in patients with anti-synthetase syndrome. Data are expressed as an absolute number (%). Ac: antibodies; ANA: antinuclear antibody.

ANA(+) (n=57)	Nuclear (N=25)	Nucleolar (N=7)	Cytoplasmic (N=37)
Myositis-specific Ac			
Anti-MDA-5	0	0	0
Anti-Mi-2	0	0	1 (2.7)
Anti-Jo-1	13 (52.0)	4 (57.1)	19 (51.4)
Anti-EJ	3 (12.0)	0	1 (2.7)
Anti-OJ	0	0	0
Anti-PL-7	4 (16.0)	2 (28.6)	4 (10.8)
Anti-PL-12	6 (24.0)	1 (14.3)	10 (27.0)
Anti-SRP	0	0	1 (2.7)
Anti-TIF-1γ	0	0	0
Anti-SAE	0	0	0
Anti-NXP-2	1/8 (12.5)	1/3 (33.3)	0
Myositis-associated Ac			
Anti-Ro-52	13 (52.0)	1 (14.3)	23 (62.2)
Anti-PM/Scl75	0	0	0
Anti-PM/Scl100	0	0	0
Anti-Ku	0	0	3 (8.1)

## Discussion

In this study, 74.4% of patients with IIM, particularly those with DM, PM, and ASyS, tested positive for ANA, most frequently displaying a nuclear pattern, followed by cytoplasmic and nucleolar patterns. MSA and MAA were more frequently observed among ANA‑positive individuals; however, this represents a descriptive distribution rather than a statistically significant difference, as group comparisons did not reach statistical significance, although some ANA-negative patients also demonstrated MSA and MAA positivity. Anti-PL-12 and -PM/Scl-100 were detected exclusively in ANA-positive individuals.

A major strength of this study was the evaluation of ANA in a representative cohort of well-defined IIM patients without concomitant systemic autoimmune diseases. Excluding overlap cases allowed ANA positivity to be attributed specifically to IIM, reducing confounding and enabling a clearer assessment of associations between ANA presence and pattern with MSA and MAA profiles.

When comparing ANA-positive and ANA-negative individuals across IIM subtypes, patients with DM, PM, and ASyS exhibited a high frequency of ANA positivity, indicating that ANA positivity is common in these subtypes, although its role should be interpreted as an association rather than a validated screening tool. In contrast, individuals with IMNM and CADM showed relatively lower ANA positivity, limiting its usefulness as a diagnostic or screening marker in these settings.

Regarding MSAs and MAAs, anti-PL-12 and -PM/Scl-100 were detected exclusively in ANA-positive patients. Conversely, the remaining autoantibodies occurred in ANA-negative individuals in up to 50% of cases, reinforcing that ANA negativity does not exclude the possibility of MSA or MAA positivity and underscoring the importance of specific autoantibody testing in clinical practice.

Among ANA-positive individuals, the most frequent co-occurring autoantibody was anti-Ro-52, identified in 50 (75.8%) of the 66 individuals who presented more than one autoantibody. Similarly, in the ANA-negative group, 16 (88.9%) of the 18 patients with multiple autoantibodies also displayed anti-Ro-52 positivity. This autoantibody is widely recognized for its association with several autoimmune diseases, including IIMs [17], and is considered a risk factor for complications such as ILD, a common manifestation within the IIM spectrum [18].

Patients diagnosed with DM who were ANA-positive predominantly exhibited autoantibodies classically associated with this subtype of autoimmune myopathy, including anti-MDA-5, -Mi-2, -TIF-1γ, -SAE, and -NXP-2 [1,3,5]. However, these autoantibodies were not exclusive: although less frequent, autoantibodies related to ASyS, such as anti-Jo-1, -EJ, -OJ, -PL-7, and -PL-12, were also observed [1,3,5]. Among ANA-positive DM patients, MAA were also identified, particularly anti-Ro-52, followed by -PM/Scl-75, -PM/Scl-100, and -Ku. Clinically, anti-Ro-52 positivity has been consistently associated with a higher frequency of ILD and worse prognosis [18]. Anti-PM/Scl-75 and -PM/Scl-100 tend to be associated with significant pulmonary and articular manifestations and higher relapse rates when they are present in patients with DM and PM [19]. Anti-Ku positivity is frequently associated with myalgia, dysphagia, and clinically meaningful proximal muscle weakness [20].

Among ANA-positive CADM patients, only a limited number of autoantibodies were detected, most notably anti-MDA-5, -TIF-1γ, -SAE, and -Ro-52, consistent with the expected serological spectrum for this subgroup [21]. However, these findings did not correlate with the ANA pattern predominantly observed in this group, as anti-MDA-5 and -Ro-52 autoantibodies do not target nuclear antigens.

ANA-positive PM patients predominantly presented MAA, anti-Ro-52, -PM/Scl-75, -PM/Scl-100, and -Ku, commonly linked to this diagnosis [22]. These autoantibodies, as in DM cases, can lead to clinical presentation that resemble ASyS; considering that anti-Ro-52 has been repeatedly linked to increased ILD rates, anti-PM/Scl-75 and -PM/Scl-100 are frequently associated with prominent joint and pulmonary involvement, in addition to higher relapse frequencies and, finally, anti-Ku is commonly related to proximal muscle weakness and dysphagia [1,3]. Nonetheless, a few MSAs were also detected, including anti-MDA-5, -Mi-2, -Jo-1, and -PL-7. Although MSA false negatives are not uncommon, some studies show that the presence of anti-Mi-2 and anti-Jo-1 in PM patients is possible and really occurs, yet the clinical outcomes and prognosis are unknown [23].

Regarding ANA-positive IMNM patients, several autoantibodies were detected, including some not typically associated with this condition, anti-MDA-5, -Mi-2, -Jo-1, -NXP-2, -Ro-52, and -Ku [1,3,5]. Nevertheless, the diagnosis was established based on clinical and laboratory characteristics and, particularly, on muscle biopsy findings, which predominantly showed macrophagic infiltration with absence of lymphomononuclear infiltrates [24]. Additionally, eight IMNM patients presented anti-SRP, a classical autoantibody associated with this subtype [1,3,5]. Anti-HMGCR was not assessed, representing a limitation, as its detection could have strengthened diagnostic accuracy in cases without the need for muscle biopsy [25]. Similar to PM patients with MSA, some IMNM patients exhibited MSA not typically associated with this subtype. Notably, IMNM has been reported in patients with isolated anti-Ku, which is consistent with our findings [26]. Moreover, histopathological features observed in ASyS may overlap with those of IMNM, potentially contributing to this serological heterogeneity [27]. Overall, the clinical and prognostic significance of these autoantibodies in IMNM remains poorly understood.

Patients with ASyS exhibited all three ANA patterns evaluated; however, the cytoplasmic pattern predominated. This predominance is consistent with the cytoplasmic localization of ARS autoantigens [28]. This finding reinforces the 2025 EULAR/ACR proposed classification criteria for ASyS [29], which emphasize the diagnostic relevance of the cytoplasmic ANA pattern. Conversely, a considerable proportion of patients did not display this ANA pattern; therefore, they will not score on this item of the proposed classification criteria. Moreover, anti-Ro-52 positivity was highly frequent among ASyS patients, a well-described characteristic of this condition [28].

A relevant limitation of this study is its retrospective design. Nevertheless, the data were obtained from electronic medical records with standardized and parameterized documentation, strengthening the reliability and robustness of the information collected. Another limitation is the long study period (2001-2025), during which classification criteria, autoantibody testing practices, and assay generations evolved. Although all samples were processed using standardized ANA interpretation and consistent line‑blot platforms available at the time of collection, temporal heterogeneity cannot be fully excluded. Moreover, the exclusion of 142 patients due to unavailable serum samples represents a substantial proportion of the screened cohort and may introduce selection bias, potentially limiting generalizability.

ANA positivity was identified in 74.4% of patients with IIM. Among these individuals, the nuclear pattern was most frequently observed in the PM, DM, CADM, and IMNM subgroups, whereas the cytoplasmic pattern predominated among AsyS patients. Most MSA and MAA autoantibodies were concentrated in the ANA-positive group, and anti-PL-12 and -PM/Scl-100 were detected exclusively in this subgroup.

## Conclusions

This study highlights that ANA testing remains a useful but limited tool in the evaluation of IIM. ANA positivity is common, particularly in DM, PM, and ASyS, and specific immunofluorescence patterns, nuclear or cytoplasmic, may provide supportive diagnostic clues aligned with underlying pathophysiology. However, the substantial heterogeneity of ANA patterns and the lower frequency of ANA positivity in subtypes such as IMNM and CADM restrict its diagnostic reliability when used in isolation.

From a clinical standpoint, the key message is that ANA negativity does not exclude the presence of MSAs and MAAs with relevant diagnostic, prognostic, and therapeutic implications. The detection of MSA and MAA in both ANA-positive and ANA-negative patients underscores the necessity of systematic, combined serological testing in suspected IIM. An integrated approach incorporating ANA interpretation, comprehensive autoantibody profiling, and clinical-phenotypic correlation is essential for accurate classification, risk stratification, and optimal management of patients with IIMs.
